# Surgical Intervention for Spinal Lesions Due to Multiple Myeloma: A Case Report

**DOI:** 10.7759/cureus.33505

**Published:** 2023-01-08

**Authors:** Metrek A Almetrek, Ahood A Mahjari, Sarah S Aldharman, Khaled A Amer, Manar F Balobaid, Abdullah Madkhali, Aisha M Alsayary, Sarah F Alsubaie

**Affiliations:** 1 Family Medicine, Ministry of Health Holdings, Abha, SAU; 2 College of Medicine, Najran University, Najran, SAU; 3 College of Medicine, King Saud Bin Abdulaziz University for Health Sciences, Riyadh, SAU; 4 College of Medicine, King Khalid University, Abha, SAU; 5 College of Medicine, Jazan University, Jazan, SAU; 6 College of Medicine, Imam Abdulrahman Bin Faisal University, Dammam, SAU

**Keywords:** saudi arbia, fractures, spinal fracture, osteolytic lesions, multiple myeloma

## Abstract

Vertebral disease is a main source of morbidity (MM) in individuals with multiple myeloma. The effects of associated osteolytic lesions and vertebral fractures on severe pain, functional limits, spinal deformity, and cord compression are well recognized. Systemic therapy, radiation, cementoplasty (vertebroplasty/kyphoplasty), and radiofrequency ablation are now available therapeutic options for severe MM spinal pain. We here reported a case of a 45-year-old male who had complained of progressive symptoms of pathological spine fractures. He had been examined and investigated for the cause of lytic lesions and found to have multiple fractures in the spine. A computed tomography (CT) revealed multiple osteolytic lesions noted in the thoracolumbar spine, ribs (bilaterally), and pelvic bones. Magnetic resonance imaging (MRI) showed a compression fracture of the T8 vertebral body with evidence of retro-bulging and a spinal canal narrowing. However, there was no evidence of spinal cord abnormal signal intensity. T2 weighted image (T2WI) keeping with edema is noted. A surgical intervention fixed the fracture and improved the quality of life. Vertebroplasty, a minimally invasive procedure, as a treatment option for vertebral lesions and pathologic fractures in the MM, showed good clinical improvement in the patient.

## Introduction

Spinal lesions are common. Although there are numerous additional etiologies, primary metastatic neoplasms are the most common cause of these lesions [1]. The most typical symptoms of these conditions include pain (local and radicular), weakness, paresthesia, and loss of bladder or bowel control. These signs indicate spinal cord compression [1]. Multiple myeloma (MM) is a neoplastic illness of B-cell origin that develops when malignant plasma cells clonally proliferate in the bone marrow [2]. The monoclonal protein, malignant cells, or cytokines produced by malignant proliferative myeloma cells cause end-organ damage such as bone destruction, pathological fractures, nephropathy, anemia, and hypercalcemia [3]. MM accounts for about 1% of all neoplastic disorders, with a median age of diagnosis around 70 years [4,5]. Bone involvement can be identified in 70-100% of patients at the time of presentation. Plasma cell invasion of the bone marrow and accelerated bone resorption induce lytic bone disease. The spine is the most prevalent location of bone localization, with 70% of patients exhibiting vertebral osteolytic lesions. In MM patients, bone involvement is the leading cause of morbidity and death [6]. Therefore, it is essential to discover the underlying cause of spinal masses and compression symptoms early on since delaying the treatment might have disastrous effects.

A recent study conducted in Saudi Arabia included 22 patients diagnosed with MM [7]. The initial symptom most frequently reported was back pain, with 204 days as the median time of diagnosis. At the time of diagnosis, the majority of patients had significant disease-related complications. This reflects the significance of recognizing the warning signs, including bone and back pain [7].

Here we present a case of symptomatic vertebral compression fractures (VCFs) in an adult patient with a newly diagnosed MM, treated with chemotherapy and bone marrow transplant, which then improved. We aimed to highlight the benefit of early treatment of this condition, which is the early consideration of the complications in order to make an early intervention at different levels of the healthcare setting.

## Case presentation

A 45-year-old Saudi man presented to the ER of Asser Central Hospital with a two-month history of sharp, progressive, non-radiating mid-right thoracic back pain without numbness or weakness in both legs. He does not have other medical diseases. The patient complained of headache and eye pain with no history of weight loss or a decrease in appetite. The patient came to the ER walking on his own without any walking aids. 

On examination, the patient was alert, conscious, ill-looking, and had a grossly intact neurological system. The patient's motor examination revealed a 5/5 strength in both upper and lower limbs. The patient was referred to a neurosurgery clinic. The neurosurgery team did general investigations that showed hypercalcemia. Table 1 shows the lab results for the patient.

**Table 1:** Lab results *Abnormal result MCV - mean corpuscular volume, MCH - mean corpuscular hemoglobin, MCHC - mean corpuscular hemoglobin concentration, CRP - C-reactive protein, ESR - erythrocyte sedimentation rate

Test	Patient results	Reference range
WBCs	16.66*	4.5-11.5 k/ul
RBCs	4	4-5.4 k/ul
Hemoglobin	10.7*	12.0-15.0 g/dL
Hematocrit	34.2*	35-49%
MCV	85.3	80-94 fL
MCH	26.7*	32-36 pg
MCHC	31.3*	32-36%
Platelets	480	150-450 k/ul
Albumin	23*	40.2-47.6 g/L
Total protein	81	64-82 g/L
CRP	3.2*	0-3 mg/L
ESR	28*	1-20 mm/H
Immunoglobulin-G	11.4	5.4-16.1 g/L
Immunoglobulin-A	1.7	0.8-2.80 g/L
Immunoglobulin-M	0.26*	0.5-1.9 g/L
Kappa	1.77	1.7-3.7 g/L
Lambda	0.99	0.9-2.1 g/L
B2-Mircoglobulin	7.9*	0.7-1.8 mg/L
Aspartate aminotransferase	8*	15-37 U/L
Calcium	9.8*	6-8 mg-Ldl
Alanine aminotransferase	19	12-78 U/L
Gamma-glutamyl transferase	3*	5-85 U/L
Bilirubin	3	0-17 umol/L
Lactate dehydrogenase	154	100-240 U/L

**Figure 1:**
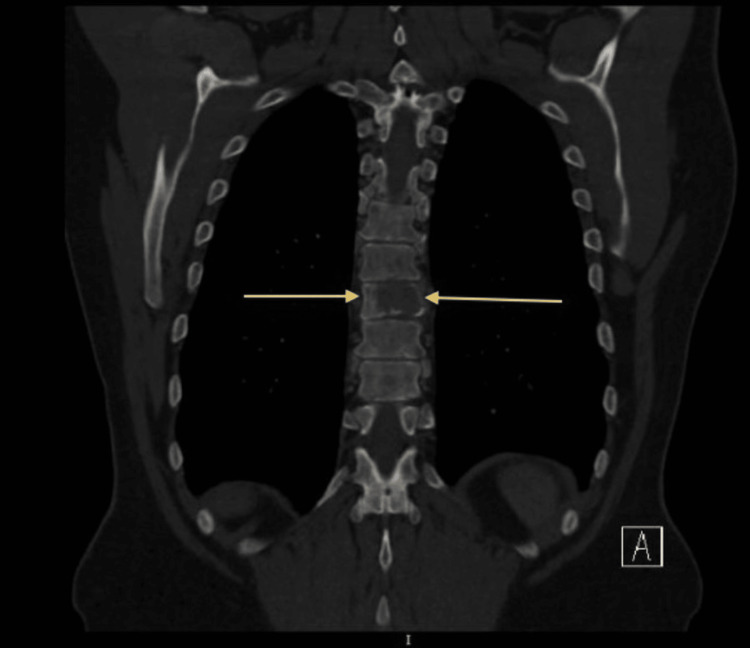
CT scan of the chest and abdomen with oral and IV contrast revealed an osteolytic lesion noted in the thoracic spine

**Figure 2:**
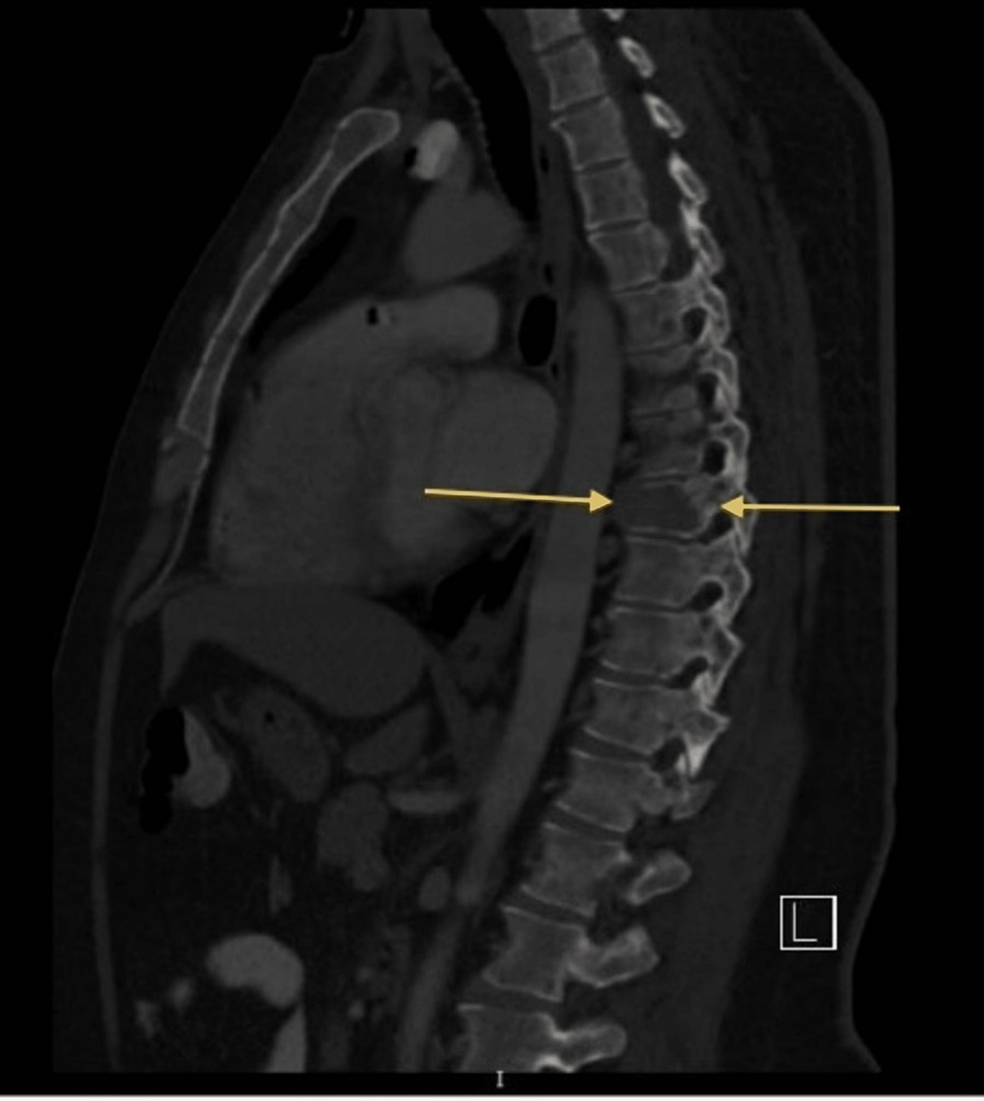
CT scan of the chest and abdomen with oral and IV contrast revealed an osteolytic lesion noted in the thoracic spine

Then they ordered a CT scan of the chest and abdomen with oral and IV contrast, which revealed multiple osteolytic lesions noted in the thoracolumbar spine and pelvic bones. One view of the CT scan revealed an osteolytic lesion noted in the thoracic spine, as shown in Figure 1. Another view of the CT scan revealing an osteolytic lesion in the thoracic spine is shown in Figure 2.

**Figure 3:**
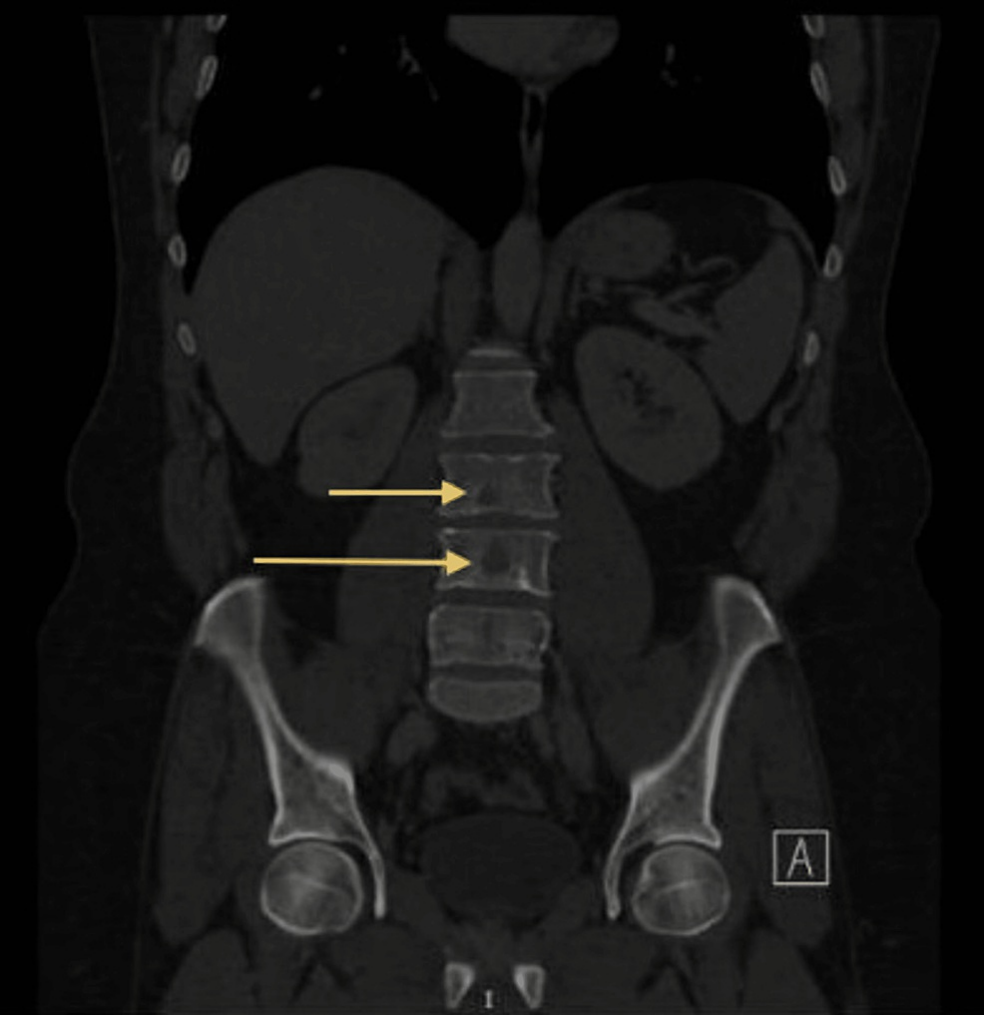
A CT scan of the chest and abdomen with oral and IV contrast, revealed multiple osteolytic lesions in the thoracolumbar spine

**Figure 4:**
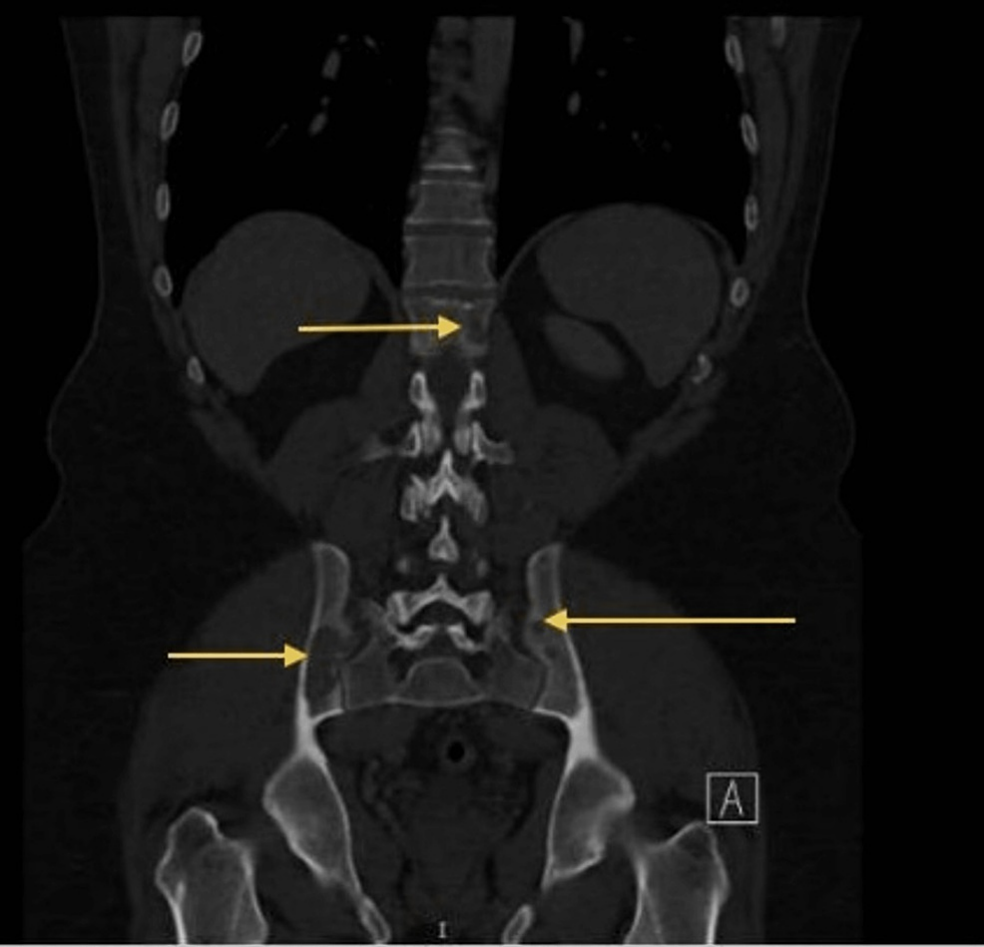
A CT scan of the chest and abdomen with oral and IV contrast, revealed multiple osteolytic lesions in the thoracolumbar spine and pelvic bones

Additional provided CT scan images in Figure 3 and Figure 4 showed multiple osteolytic lesions in the thoracolumbar spine and pelvic bones.

T8 vertebral body showed an expansile osteolytic lesion with cortical destruction and extension into the paraspinal area and posteriorly into the spinal canal, compressing the thecal sac. T8 vertebral height was diminished, indicating a compression fracture. An isotope bone scan and tissue biopsy were advised for further evaluation.

**Figure 5:**
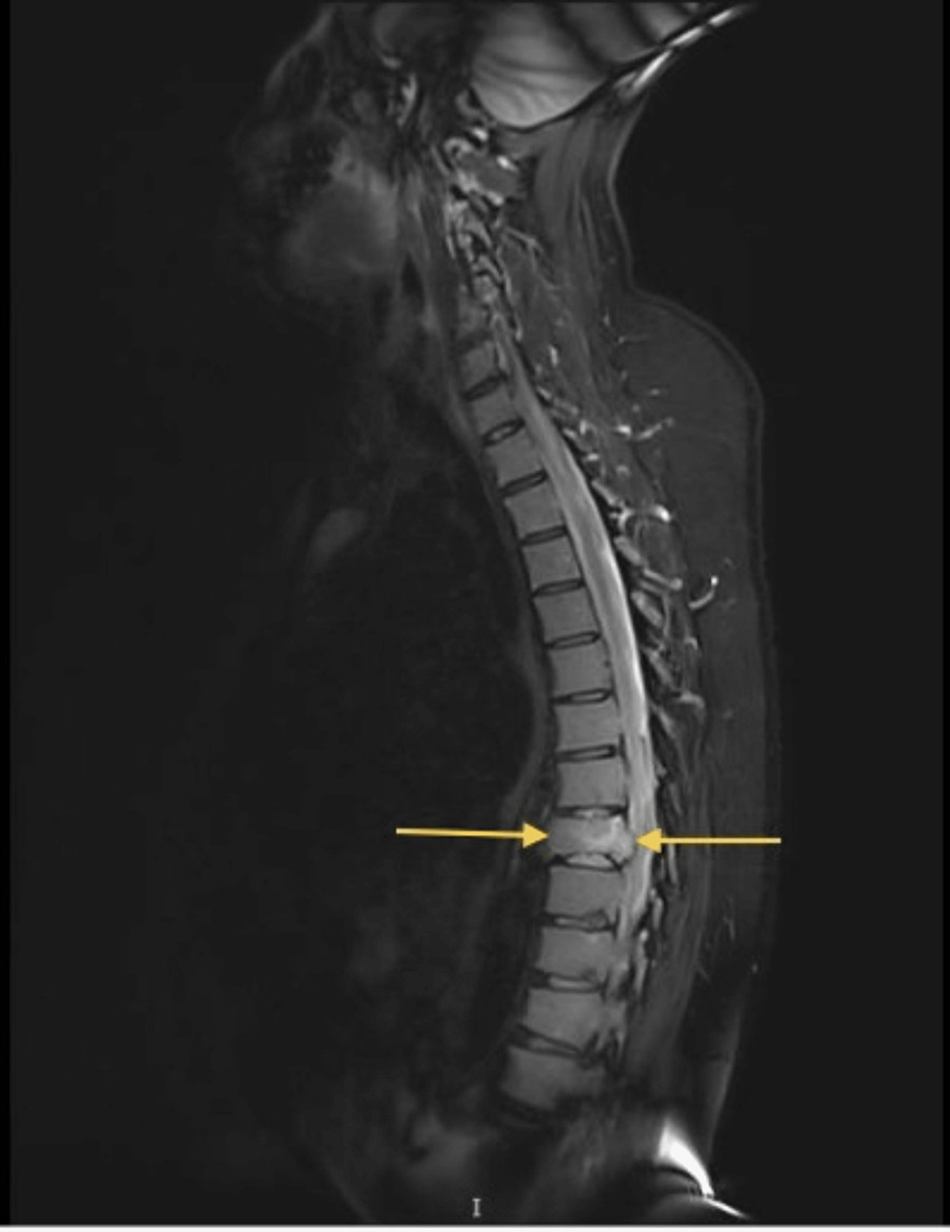
A spine MRI without contrast revealed a compression fracture of the T8 vertebral body with evidence of retro-bulging and a spinal canal narrowing

A spine MRI was planned which revealed a compression fracture of the T8 vertebral body with evidence of a retro-bulging and a spinal canal narrowing. However, there was no evidence of spinal cord abnormal signal intensity. T2WI keeping with edema is noted. A spine MRI without contrast revealed a compression fracture of the T8 vertebral body with evidence of retro-bulging and a spinal canal narrowing, as shown in Figure 5.

**Figure 6:**
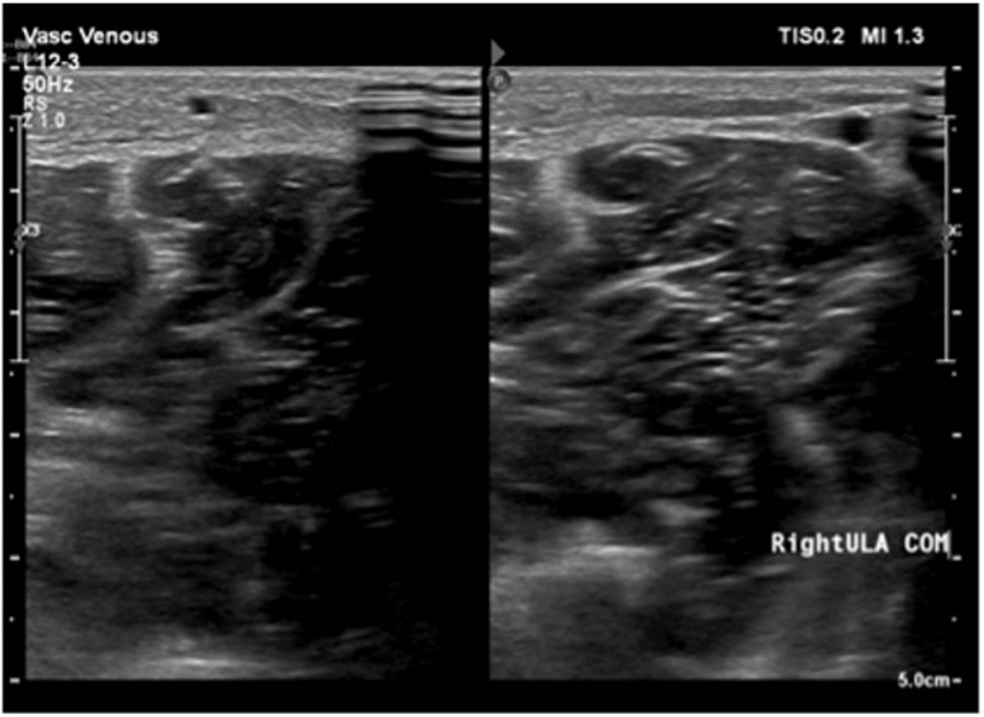
A venous doppler of the right upper limb showed a dilated non-compressible median cubital vein extending to a cephalic vein with echogenic thrombus totally occluded its lumen with no color flow and doppler spectral waves at its course in the forearm

The patient started to complain of right upper limb swelling and redness; therefore, a venous doppler of the right upper limb was ordered. The test revealed normal compressibility and color flow within the right subclavian, right axillary, and right brachial veins, with no evidence of recent deep venous thrombosis. Moreover, the test also showed a dilated non-compressible median cubital vein extending to the cephalic vein with echogenic thrombus totally occluded its lumen with no color flow and doppler spectral waves at its course in the forearm. These findings are suggestive of right upper limb thrombophlebitis. The venous doppler of the right upper limb is shown in Figure 6. 

The neurosurgery department in Asser Hospital did vertebroplasty for the T8 vertebral body, and a bone biopsy of the T8 vertebral body was obtained by vertebroplasty needle during the same procedure. The patient's low back pain was relieved. On histological examination, the obtained specimen revealed infiltration of atypical plasma cells. Immunohistochemical analysis revealed that the plasma cells expressed CD56 and demonstrated monotypic expression of lambda light chain. The internal medicine department in Asser Central Hospital started anti-inflammatory, anticoagulant, and antibiotic drugs as supportive treatments to avoid any complications. The neurosurgery department referred the patient to a higher center for oncology hematology in King Faisal Specialized Hospital and Research Center (KFSH&RC). KFSH&RC started the vincristine, Adriamycin, and dexamethasone (VAD) chemotherapy four times, along with a bone marrow transplantation. The M-spike then decreased from 1.10 to 0.22 g/dL on serum protein electrophoresis. The results of protein electrophoresis are shown in Table 2. The patient is still alive without developing symptoms one year after the diagnosis of multiple myeloma. 

**Table 2:** Protein electrophoresis at the time of initial diagnosis *Abnormal result

Test	Patient results	Reference range
Albumin	32.2*	40.2-47.6 g/l
Alpha-1	5.4*	2.1-3.5 g/l
Beta-2	4	2.3-4.7 g/l
Gamma	12.6	8.0-13.5 g/l

## Discussion

Multiple myeloma is a neoplastic disorder of plasma cells, which accounts for 10% of hematological malignancies, making it the second most common hematological malignancy. Risk factors for this malignancy are yet to be identified, but its incidence is higher in males and African ethnicity [8-10]. One of the most common presentations of this malignancy is back pain, which occurs in up to 58% of patients. Lytic lesions are one of the early findings detectable in almost 80% of patients at the time of diagnosis. Early recognition of the disease's characteristics allows for early management, leading to an improvement in the patient's quality of life [11].

Here we present a case of a newly diagnosed multiple myeloma patient who presented with back pain which was then attributed to a spinal compression fracture. After further evaluation, the neurosurgery department detected multiple lytic lesions involving the thoracolumbar spine, ribs (bilaterally), and pelvic bones. The neurosurgery team managed the compression fracture by vertebroplasty, which led to a good outcome and an alleviation of the patient's back pain.

The American Journal of Hematology published the 2022 update on the diagnosis of multiple myeloma [12], which supports the diagnosis of multiple myeloma for this patient. The combination of chronic back pain, anemia, high total protein, low albumin, hypercalcemia, an abnormal increase of gamma protein in protein electrophoresis along with the compression fracture of the T8 vertebra in MRI all support the diagnosis of multiple myeloma. The neurosurgery team managed the back pain with vertebroplasty, which proved to be effective in relieving the pain rapidly and significantly, thus, reducing the use of analgesics.

In a retrospective view of clinical data from 67 multiple myeloma patients who were treated with vertebroplasty, the results showed that 89% of those patients had experienced significant alleviation of the pain, and 65% of them needed fewer narcotics for pain management after the procedure [13]. The surgical treatment of pathological spinal fractures, which are associated with multiple myeloma, is by augmentation surgeries such as percutaneous vertebroplasty or balloon kyphoplasty. Both surgeries give patients a better outcome compared to conservative management. A similar case reported multiple compression fractures in a newly diagnosed multiple myeloma (MM) patient, in which they treated the patient with balloon kyphoplasty, which resulted in a significant alleviation of the patient's pain [6]. KFSH&RC had started the patient on combination chemotherapy as an induction therapy before stem cell transplantation, which can lead to an improved outcome [14].

One of the most frequent locations for the initial manifestation of MM metastases is the spine, accounting for 80% of metastatic bone lesions [15]. Given how common low back pain is in Saudi Arabia, it is not surprising that multiple myeloma diagnosis is frequently missed, even though it may be the earliest indicator of malignancy [16,17]. A single clone of plasma cells produced from B cells that are proliferating in the bone marrow is the hallmark of multiple myeloma. The adjacent bone is frequently invaded, which destroys the skeletal components causing bone pain and fractures [18]. Bone lesions can affect any part of the skeleton, including the spine, skull, and long bones. The primary mechanism of MM bone disease is increased osteoclastogenesis with reduced osteoblastic activity [19]. According to recent papers, the receptor activator of nuclear factor kappa-B ligand (RANKL) and osteoprotegerin (OPG) mechanism is key in this regard [20].

MM is considered to be the second most common hematologic malignancy after non-Hodgkin's lymphoma. This article reports the case of a 45-year-old Saudi Arabian man with MM, osteolytic lesions, and vertebral fractures. To our knowledge, this is the first case in Saudi Arabia discussing the surgical intervention for spinal lesions for multiple myeloma. Because MM is highly sensitive to radiotherapy, a few studies in the literature recommend combined radiotherapy with high-dose steroids as first-line therapy [21,22]. In contrast, several reports have attempted surgical decompression and recommended early surgical decompression followed by radiotherapy or chemotherapy [23-26]. Our patient underwent chemotherapy and bone marrow transplantation and subsequently was improved, but the hospital's neurosurgeons performed vertebroplasty of the T8 vertebra, and during the vertebroplasty, a bone biopsy was obtained. The patient's lower back pain was relieved. The internal medicine department at Aseer Hospital initiated anti-inflammatory, anticoagulant, and antibiotic medications to prevent complications. The patient received four rounds of VAD chemotherapy, and he underwent a bone marrow transplant. M-spike subsequently decreased from 1.10 to 0.22 g/dL by serum protein electrophoresis. To evaluate the vertebroplasty procedure, Table 3 shows the existing literature on vertebroplasty outcomes in patients with multiple myeloma.

**Table 3:** Vertebroplasty and its outcomes in patients with multiple myeloma VAS - Huskisson's visual analog scale, MGM - McGill-Melzack scoring system, PV - percutaneous vertebroplasty, SF-36 - 36-Item Short Form Health Survey

Study Title	Outcome
Prospective evaluation of pain relief in 100 patients undergoing percutaneous vertebroplasty: results and follow-up [27]	The operation was successful in all patients. However, there were two complications. One patient sustained a sternal fracture and one experienced transient radiculopathy. 97% reported pain relief 24 hours post-treatment. The mean follow-up duration was 21.5 months in 99 patients. 93% reported significant relief in back pain previously associated with their compression fractures as well as improved movement and ambulatory ability. Before vertebroplasty, the VAS score for the 99 patients was 8.91 +/- 1.12 compared to a score of 2.02 +/- 1.95 at follow-up postoperatively.
Prospective clinical follow-up after percutaneous vertebroplasty in patients with painful osteoporotic vertebral compression fractures [28]	After percutaneous vertebroplasty (PV), a significant reduction in VAS scores for pain at the individual vertebral levels treated and the use of analgesic agents was noticed compared with before treatment at every follow-up period. The preprocedural mean VAS score was 8.8 (range, 5-10), while at follow-up, mean VAS scores ranged from 2.5 to 3.3 (range, 0-10). In the short-term post-PV, patients used significantly fewer analgesic drugs and 86% of patients were satisfied with the outcome. At midterm and long-term follow-ups, patients used even fewer analgesic drugs and 95-100% of patients were satisfied with the outcome of PV. Procedure-related complications with clinical consequences occurred in three patients: one patient experienced a cardiovascular reaction, one patient had a pedicle chip fracture, and one had a rib fracture.
Prospective analysis of clinical outcomes after percutaneous vertebroplasty for painful osteoporotic vertebral body fractures [29]	Pre- and post-treatment pain scores were 8.71 (SE=0.1) and 2.77 (SE=0.18; p<.00001) respectively pre and post-treatment analgesic use scores were p activity levels statistically significant improvement on nine of sf-36 scales after one month eight at long-term follow-up was observed.
Long-term follow-up of vertebral osteoporotic fractures treated by percutaneous vertebroplasty [30]	The mean duration of follow-up was 35 months. Pain assessed by the VAS significantly decreased from a mean of 71.4 mm+/-13 before PV to 36 mm+/-30 after six months, and to 39 mm+/-33 at the time of maximal follow-up (p<0.05 for both comparisons). The results were also significant for the MGM: 3.00+/-0.57 before PV to 1.6+/-1.4 at the long-term follow-up (p<0.05). No severe complication occurred immediately after PV.
Medium-term results of percutaneous vertebroplasty in multiple myeloma [31]	The mean age of the participants was 66 years. Significant improvement occurred one day after the procedure according to the VAS score (7.5 pre-PV to 3.7, p< 0.0001). This significant improvement was maintained after 3.2 years of follow-up. 63% of patients were highly satisfied with the result of the procedure and 37% were satisfied. The peri-operative mortality was 0%. Leakage of the cement outside of the vertebral body was noted in 16 of 19 injected vertebrae (84%) but none of the patients developed any clinical or neurological symptoms. At the last follow-up, no further collapse in the treated or neighboring vertebrae was noted. Percutaneous vertebroplasty is associated with early clinical improvement of pain and function and can be maintained after a long follow-up without major procedure-related complications.

## Conclusions

Management of multiple myeloma spine metastases is an example of an interprofessional, multidisciplinary, and comprehensive approach. Early diagnosis is the mainstay for a favorable outcome after the treatment. Vertebroplasty, as indicated, should be performed as early as possible. Vertebroplasty improves the quality of life, gives vital outcomes, and minimizes complications. Spinal cord compression is one of the worst side effects of multiple myeloma, and the best therapeutic approach is still up for debate. The authors attempted to offer a therapy plan for the best clinical outcomes by reviewing cases with various clinical courses and pertinent literature. Close examination of the mechanical stability and neurologic condition, as well as a multidisciplinary approach, are crucial for effective outcomes in this systemic disease.

## References

[1] Chakraborti C, Miller KL (2010). Multiple myeloma presenting as spinal cord compression: a case report. J Med Case Rep.

[2] Kyle RA, Rajkumar SV (2004). Multiple myeloma. N Engl J Med.

[3] Kumar SK, Callander NS, Adekola K (2020). Multiple myeloma, version 3.2021, NCCN Clinical Practice Guidelines in Oncology. J Natl Compr Canc Netw.

[4] Siegel RL, Miller KD, Fuchs HE, Jemal A (2021). Cancer statistics, 2021. CA Cancer J Clin.

[5] Vincent Rajkumar S (2014). Multiple myeloma: 2014 Update on diagnosis, risk-stratification, and management. Am J Hematol.

[6] Giorgi PD, Schirò GR, Capitani D, D'Aliberti G, Gallazzi E (2020). Vertebral compression fractures in multiple myeloma: redefining the priorities during the COVID-19 pandemic. Aging Clin Exp Res.

[7] Elghazaly A, AlSwayyan A, AlGreshah H (2020). Impact of delayed diagnosis of multiple myeloma. J Appl Hematol.

[8] Rajkumar SV (2020). Multiple myeloma: 2020 update on diagnosis, risk-stratification and management. Am J Hematol.

[9] Alexanian R, Dimopoulos M (1994). The treatment of multiple myeloma. N Engl J Med.

[10] Heider M, Nickel K, Högner M, Bassermann F (2021). Multiple myeloma: molecular pathogenesis and disease evolution. Oncol Res Treat.

[11] Eslick R, Talaulikar D (2013). Multiple myeloma: from diagnosis to treatment. Aust Fam Physician.

[12] Rajkumar SV (2022). Multiple myeloma: 2022 update on diagnosis, risk stratification, and management. Am J Hematol.

[13] McDonald RJ, Trout AT, Gray LA, Dispenzieri A, Thielen KR, Kallmes DF (2008). Vertebroplasty in multiple myeloma: outcomes in a large patient series. AJNR Am J Neuroradiol.

[14] Kumar S, Giralt S, Stadtmauer EA (2009). Mobilization in myeloma revisited: IMWG consensus perspectives on stem cell collection following initial therapy with thalidomide-, lenalidomide-, or bortezomib-containing regimens. Blood.

[15] Lote K, Walløe A, Bjersand A (1986). Bone metastasis. Prognosis, diagnosis and treatment. Acta Radiol Oncol.

[16] Alnaami I, Awadalla NJ, Alkhairy M (2019). Prevalence and factors associated with low back pain among health care workers in southwestern Saudi Arabia. BMC Musculoskelet Disord.

[17] Mabry LM, Ross MD, Tonarelli JM (2014). Metastatic cancer mimicking mechanical low back pain: a case report. J Man Manip Ther.

[18] Kyle RA, Rajkumar SV (2009). Criteria for diagnosis, staging, risk stratification and response assessment of multiple myeloma. Leukemia.

[19] Bataille R, Chappard D, Marcelli C, Dessauw P, Sany J, Baldet P, Alexandre C (1989). Mechanisms of bone destruction in multiple myeloma: the importance of an unbalanced process in determining the severity of lytic bone disease. J Clin Oncol.

[20] Vanderkerken K, De Leenheer E, Shipman C, Asosingh K, Willems A, Van Camp B, Croucher P (2003). Recombinant osteoprotegerin decreases tumor burden and increases survival in a murine model of multiple myeloma. Cancer Res.

[21] Prestwich RJ, Ackroyd S, Gilson D (2011). Is surgery required in the management of spinal cord compression in myeloma patients?. Clin Oncol (R Coll Radiol).

[22] Jin R, Rock J, Jin JY, Janakiraman N, Kim JH, Movsas B, Ryu S (2009). Single fraction spine radiosurgery for myeloma epidural spinal cord compression. J Exp Ther Oncol.

[23] Dürr HR, Wegener B, Krödel A, Müller PE, Jansson V, Refior HJ (2002). Multiple myeloma: surgery of the spine: retrospective analysis of 27 patients. Spine (Phila Pa 1976).

[24] Lourbopoulos A, Ioannidis P, Balogiannis I, Stavrinou P, Koletsa T, Karacostas D (2011). Cervical epidural plasmacytoma presenting as ascending paraparesis. Spine J.

[25] Okacha N, Chrif E, Brahim E (2008). Extraosseous epidural multiple myeloma presenting with thoracic spine compression. Joint Bone Spine.

[26] Renier JC, Brégeon C, Boasson M (1984). Spinal cord compression in multiple myeloma. Study of 10 cases (Article in French). Rev Rhum Mal Osteoartic.

[27] McGraw JK, Lippert JA, Minkus KD, Rami PM, Davis TM, Budzik RF (2002). Prospective evaluation of pain relief in 100 patients undergoing percutaneous vertebroplasty: results and follow-up. J Vasc Interv Radiol.

[28] Voormolen MH, Lohle PN, Lampmann LE, van den Wildenberg W, Juttmann JR, Diekerhof CH, de Waal Malefijt J (2006). Prospective clinical follow-up after percutaneous vertebroplasty in patients with painful osteoporotic vertebral compression fractures. J Vasc Interv Radiol.

[29] Do HM, Kim BS, Marcellus ML, Curtis L, Marks MP (2005). Prospective analysis of clinical outcomes after percutaneous vertebroplasty for painful osteoporotic vertebral body fractures. AJNR Am J Neuroradiol.

[30] Legroux-Gérot I, Lormeau C, Boutry N, Cotten A, Duquesnoy B, Cortet B (2004). Long-term follow-up of vertebral osteoporotic fractures treated by percutaneous vertebroplasty. Clin Rheumatol.

[31] Ramos L, de Las Heras JA, Sánchez S, González-Porras JR, González R, Mateos MV, San Miguel JF (2006). Medium-term results of percutaneous vertebroplasty in multiple myeloma. Eur J Haematol.

